# Lichenoid Keratosis Skin Biopsy: A Case Report of Malignant Hospital Charges

**DOI:** 10.7759/cureus.13292

**Published:** 2021-02-11

**Authors:** John R. Adler, Hong Do, Jeffrey Pfeffer

**Affiliations:** 1 Department of Neurosurgery, Stanford University School of Medicine, Stanford, USA; 2 Internal Medicine/Dermatology, Regional Medical Center, San Jose, USA; 3 Stanford Graduate School of Business, Stanford University, Stanford, USA

**Keywords:** lichenoid keratosis, skin biopsy, hospital charges

## Abstract

Skin cancers are the most common malignancy and are especially common among light-skinned individuals in sun-exposed areas. While in many cases, a characteristic or classic appearance of the lesion is sufficient to make a definitive diagnosis, shave biopsy remains an important procedure when diagnosing many such raised lesions. Over the span of two months, a 66-year-old Caucasian male noted the appearance of a small, raised pruritic scaly lesion over his right upper chest. The differential diagnosis included both cancerous and benign lesions. During a 15-minute clinic visit, a simple shave biopsy was performed, and additionally, 10 small actinic keratoses on the patient’s arms, legs, and back were treated with cryotherapy using liquid nitrogen. Later, a histologic examination of the biopsied lesion revealed a benign lichenoid keratosis. The patient was billed $10,187 for this outpatient experience.

## Introduction

Cutaneous basal and squamous cell carcinoma are exceedingly common malignancies, especially in the sun-exposed areas of light-skinned individuals. Recently appearing raised, reddish, scaly, and/or shiny lesions in sun-damaged skin are pathognomonic of skin cancer. However, the appearance of sundry benign lesions occurring in the same locations can mimic cancer, so biopsy and histologic confirmation is often necessary. The virtue of a shave biopsy is that it may be diagnostic, less invasive, and curative in the case of most skin cancers. Generally speaking, a cutaneous shave biopsy is among the very simplest of all operative procedures, requiring minimal surgical facilities and technical skills. However, the collective management of skin cancer, which consists largely of surgical resection, is among the most expensive of all cancers for society [1].

What should be the proper “cost” of a healthcare service? This challenge continues to plague the healthcare system worldwide. In the US, a common term of art among government and private insurance companies is “Usual, Customary and Reasonable” (UCR) Fees. However, after accounting for such, the charges associated with specific medical procedures vary considerably between different US hospitals and facilities, so much so that noted Princeton healthcare economist Uwe Reinhardt referred to them as the price “a drunken billionaire would pay a hospital if his wife were not around to control the bastard” [2]. Egregious healthcare charges have been well-documented in the news media, usually originating from anonymous healthcare facilities and without making an effort to benchmark the extent of “overbilling.” In this report, we seek to compare a rather simple real-world medical bill against what other US and international healthcare providers might have charged for a comparable procedure.

## Case presentation

**Figure 1:**
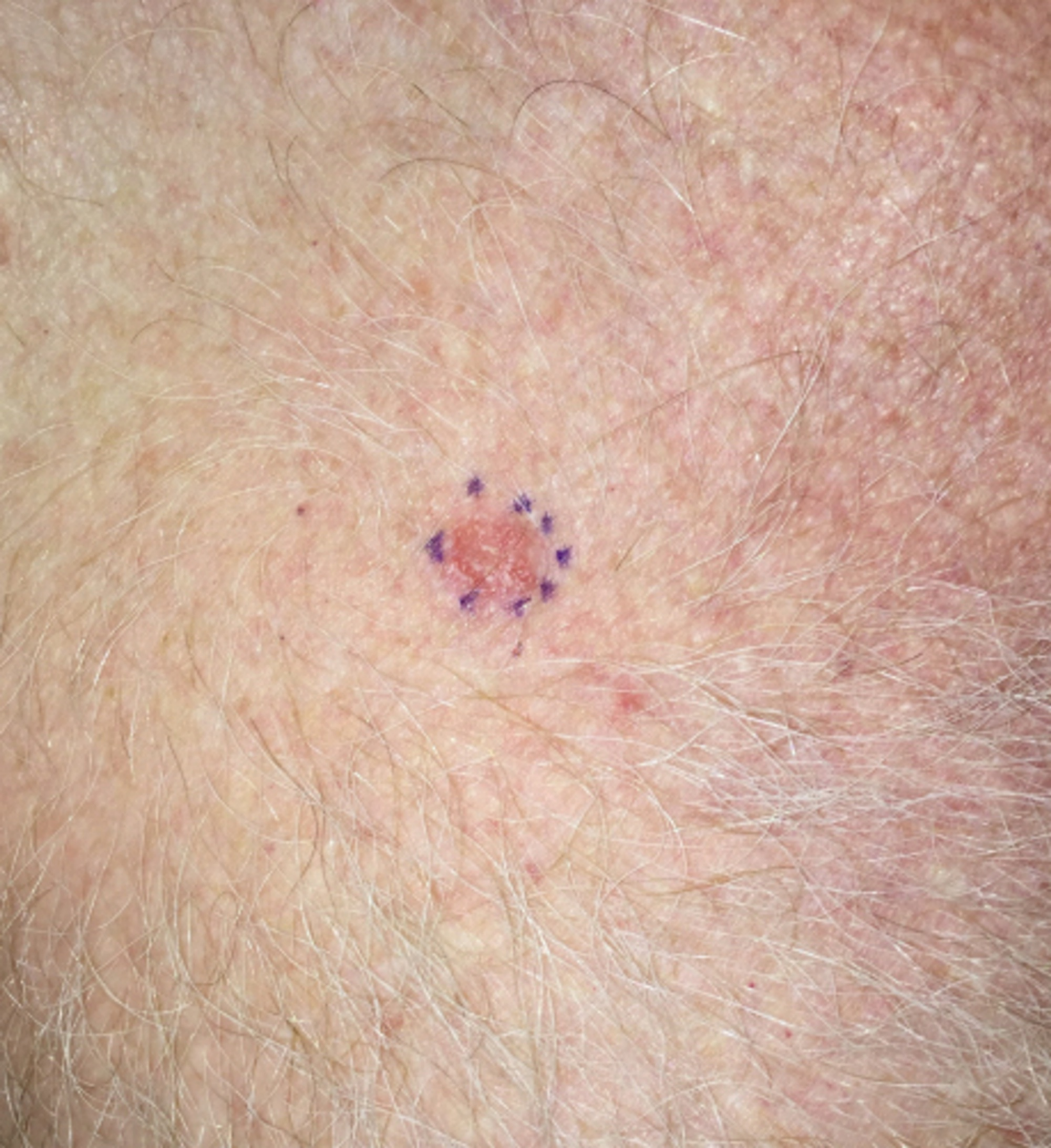
Chest wall lesion Photograph of skin lesion outlined by a blue marker pen prior to the shave biopsy

A 66-year-old, generally healthy Caucasian male, photo skin type with red hair, noted over the span of two months the appearance of a 1 x 1 cm, scaly, pruritic, right anterior subclavicular chest wall skin lesion (Figure 1).

**Figure 2:**
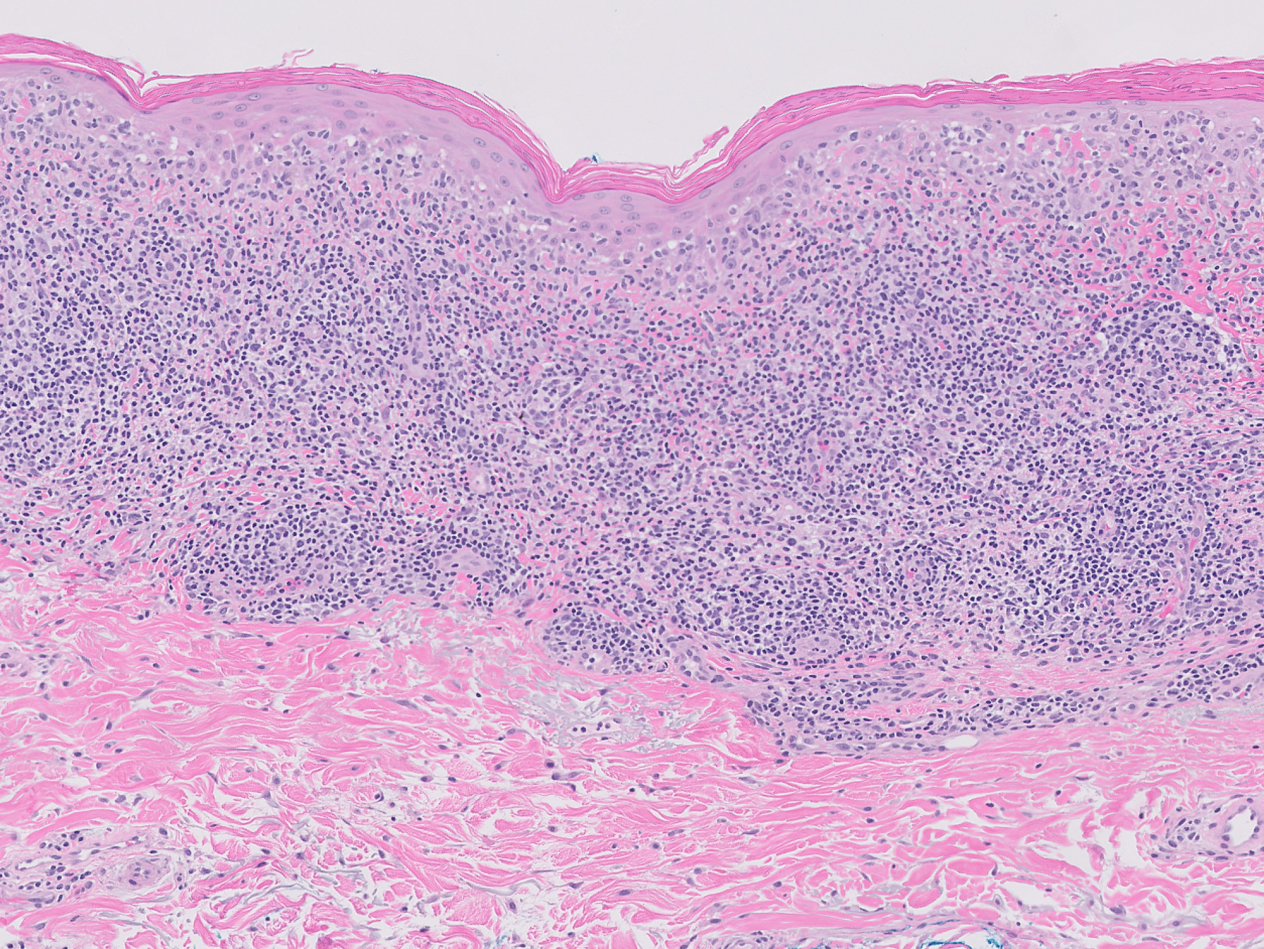
Photomicrograph after hematoxylin and eosin (H&E) staining Note that the lesion displayed the classic histologic appearance of lichenoid keratosis with a band-like infiltrate of lymphocytes filling the papillary dermis. In addition, there are lichen planus-like changes at the junction between the epidermis and dermis with both vacuolar alteration of the basal layer and necrotic keratinocytes (Civatte bodies).

Based on visual inspection, the differential diagnosis included squamous cell cancer (SCC), hypertrophic actinic keratosis (HAK), and irritated seborrheic keratosis (ISK). To make a definitive diagnosis, a biopsy was recommended and the patient consented. After subcutaneous instillation of 1% xylocaine local anesthesia, a straightforward, uncomplicated shave biopsy was performed. The 1.3 x 1.0 x 0.2 (depth) cm biopsied lesion was fixed in formalin. In addition, 10 actinic keratoses on the arms, face, and legs, each measuring less than 5 mm in diameter, were treated with cryotherapy using liquid nitrogen. The entire procedure time (shave biopsy and cryoablation) lasted less than 10 minutes. The subsequent pathologic report indicated that the excised lesion was a lichenoid keratosis. Subsequent pathologic study established a benign diagnosis of lichenoid keratosis (Figure 2).

**Figure 3:**
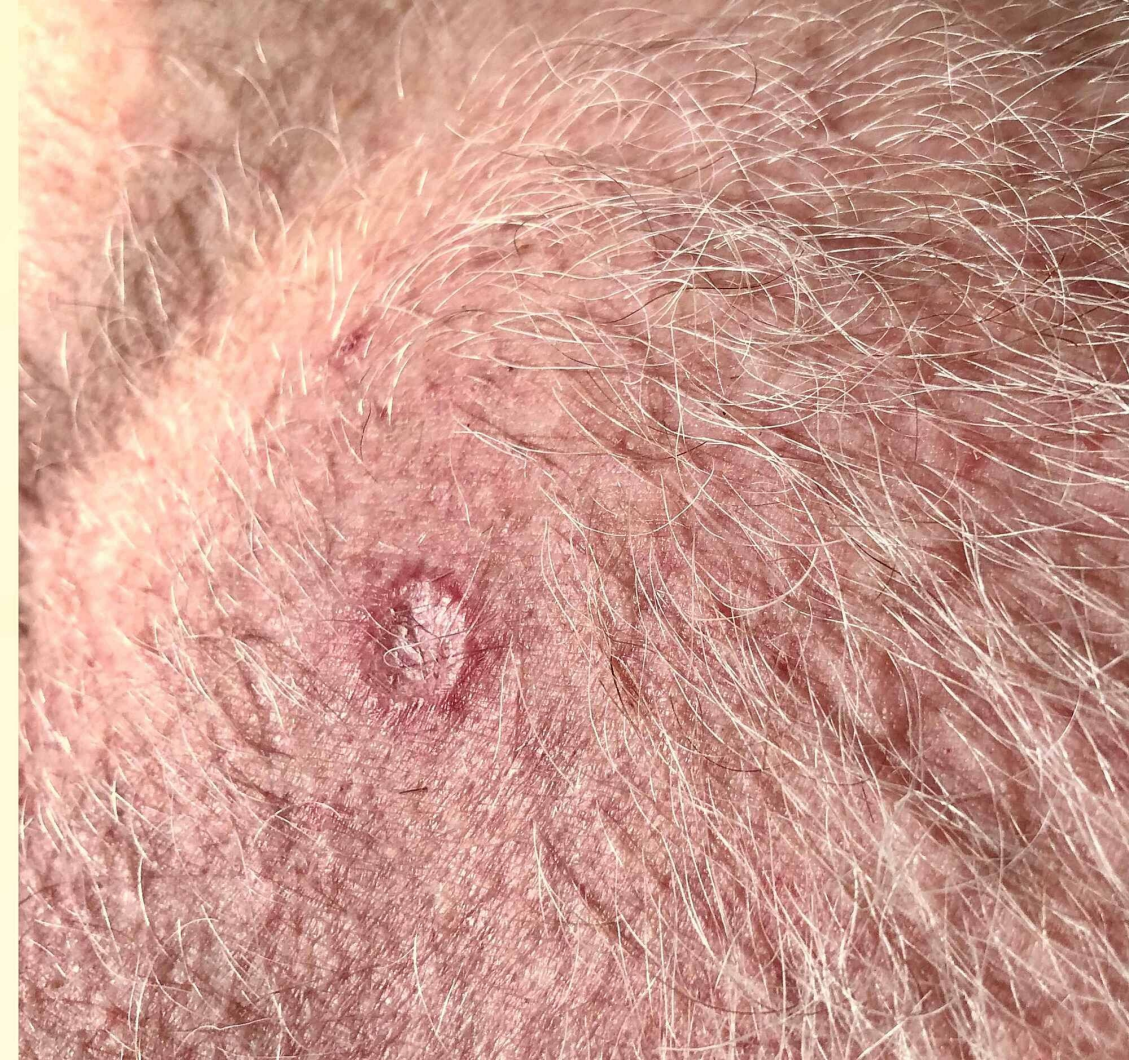
Healed skin biopsy site

At six weeks of follow-up, the skin biopsy site was healed (Figure 3).

**Figure 4:**
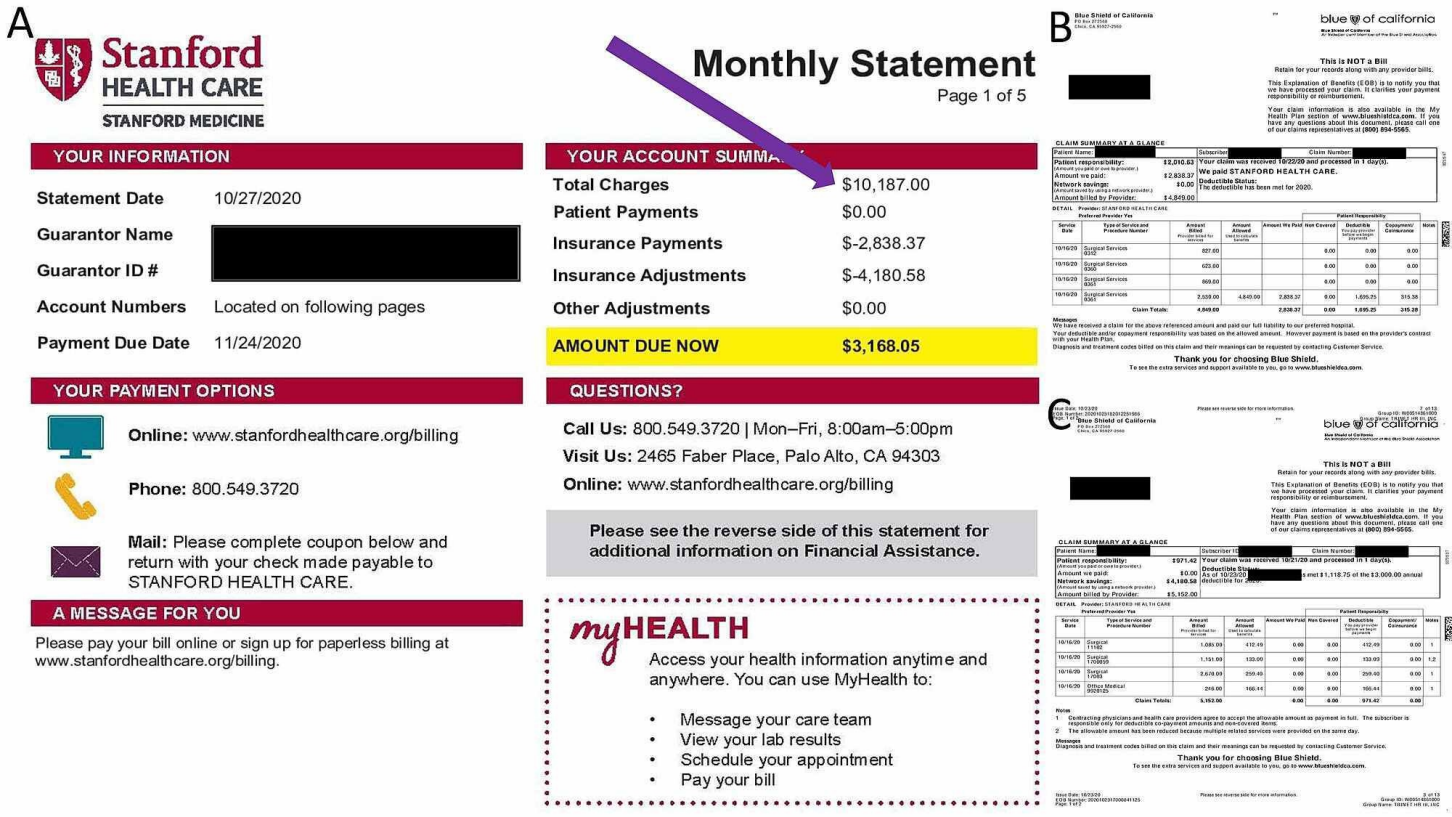
4A) Hospital billing statement, 4B) Estimation of Benefits (EOB) for cryosurgery, 4C) EOB for a shave biopsy Note total charge of $10,187.

The hospital, a leading American academic medical center, which was ranked by US News and World Report as a top 20 medical center, billed the patient $10,187 in total charges for this outpatient procedure (Figures 4A-4C).

**Figure 5:**
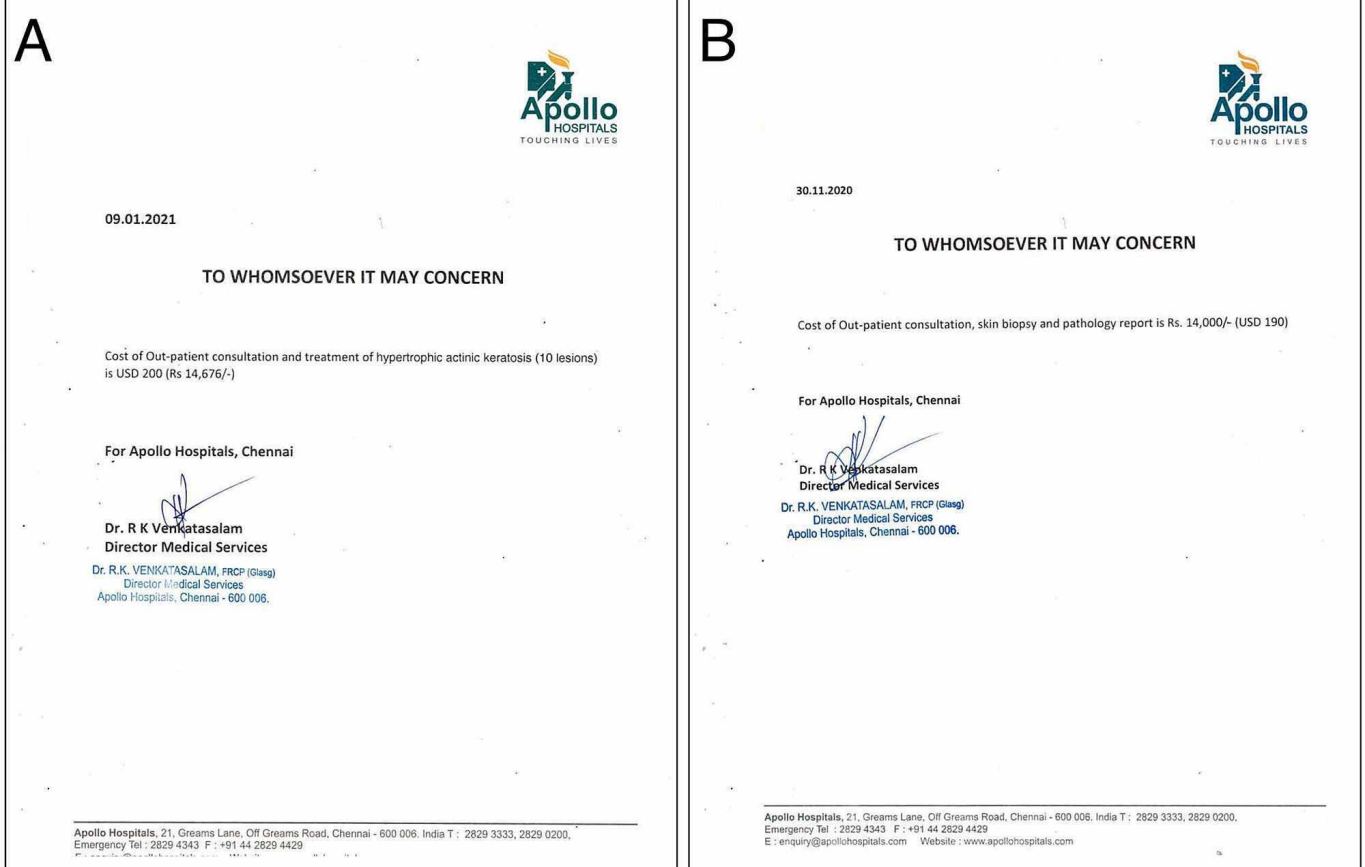
Apollo Hospital quotes for A) Cryoablation, B) Shave biopsy Provided by Apollo Hospital's Health Tourism Office in Chennai, India

The above Estimation of Benefits (EOB) was shared with a private practice dermatologist (Hong Do, MD) in Santa Clara County, CA (the same county as Stanford Medical Center) and the Health Tourism Team at Apollo Hospital in Chennai, India, which like all eight Apollo hospitals, is accredited by the Joint Commission International (JCI). Quotes for the same level of care as administered at Stanford Medical Center were generated (Figures 5A, 5B).

Table 1 summarizes all the prices.

**Table 1:** Summary of all hospital charges and comparable estimated charges

EOB	Stanford Medical Center	SCC Private Practice	Apollo Hospital Chennai, India
Shave biopsy with pathology (11102, 17059, 17003, 99201, 88305)	4,849 USD	1,020 USD	190 USD
Destruction of HAK x 10 (17000, 17110)	5,338 USD	320 USD	200 USD
TOTAL	10,187 USD	1,340 USD	390 USD

## Discussion

In this anecdotal report, we present a rather ordinary case of skin lesions successfully managed with commonplace dermatological procedures. The clinical presentation and outcome in this patient are utterly unremarkable. In fact, we publish this case not because the clinical dimension is unique but because it is so thoroughly ordinary. In this case, it is easy to argue that no special (tertiary) medical expertise was required to administer quality care. Equally importantly, because the patient in question involved a co-author, someone who could readily waive all HIPAA rights, it was possible to more simply convey financial facts that otherwise constitute heavily regulated (by US law) personal identifying information.

The striking finding in the case we present is the relatively huge difference in charges between the different clinical settings. The quote from Apollo, an Indian private hospital, illustrates, somewhat arguably, the base cost of providing rudimentary care, which, in part, factors the cost of essential medical supplies whose prices are largely controlled by supply and demand in an international marketplace. Labor costs are obviously significantly different between the US and Chennai. In contrast, the detailed procedural quote using standardized EOBs from the same county as Stanford Medical Center (Santa Clara County in California) should be representative of the regional costs of the same procedure in question, including labor. Ultimately, what we see is that charges at Stanford Medical Center range (roughly) between 10X and 20X the cost of near-identical services provided in a US-based private practice or in a low-cost international medical facility, respectively. Importantly, higher costs are not about better quality, which is what the present “anecdote” seeks to possibly illustrate. Even if one rejects this assertion about quality, it seems absurd to suggest that healthcare quality for the procedure in question is 10X or 20X better than the care delivered inside a private practice and/or high-quality Indian hospitals!

Given the above context, the important “story” we seek to tell in this case report is obviously unrelated to the clinical circumstances. This is a story about hospital charges billed for a relatively minor procedure, yet not just by any hospital, but a major American academic medical center. One might argue that the “charges” in question are a mere anecdote involving a single procedure at a single US academic medical center. However personal experience over a 30-year medical career and many news media reports tell a different story [3]. High charges are the norm among hospitals, in general, but especially within US academic institutions, as seen with this case.

Although self-perceived to deliver uniquely high-quality care, academic medical centers are among the costliest facilities in America to receive healthcare [4]. US-based academic medical centers (AMCs) often ascribe their higher charges to the cost of post-graduate training, despite being separately paid by Medicare for such educational services or approximately $112,000 per year, per resident [5]. Notably, these institutions get paid four times for their services: through charges for clinical care, government reimbursement for resident training, philanthropic donations, and research grants. AMCs often explain their commitment to translational medical research as another justification for significantly higher charges. Without in-depth accounting, it is impossible to confirm this contention. Moreover, there is evidence that translational research within AMCs is not straightforward and suffers from several challenges peculiar to such often large and bureaucratic hospitals [6]. Moreover, the lead author of the current article, a lifelong academic physician and medical device entrepreneur, has observed little correlation between the higher costs of academic medical centers and true translational research, i.e. research that spans the “bench to bedside” divide, with a deliberate focus on actually making it to the patient bedside. The contention that AMCs serve a unique (and very expensive) translational medical function within healthcare could be subject to confirmation bias and would, therefore, seem to merit greater study.

At the current moment in US history, and perhaps amplified by the coronavirus disease 2019 (COVID-19) pandemic, there has been a dramatic focus throughout American society on healthcare disparities. Medical schools and their closely allied medical centers purport to be leaders in driving fairness among all their stakeholders in healthcare, especially among racial minorities and other communities that have faced historic discrimination. Therefore, it is more than ironic that the same institutions that loudly proclaim their commitment to inclusion also create gross financial barriers that prevent all but the wealthiest uninsured (and even sometimes the insured) patients from accessing medical care [7]. The cost of healthcare delivered by AMCs is at odds with the foundational principles of equity and inclusion and would seem to undermine their core medical education mission.

The problems posed by “excessive” AMC hospital charges extend far beyond the individual patients being cared for within. The faculty working inside AMCs tend to dominate healthcare discussions and many positions of medical leadership within the US government, not infrequently shuttling between academia and government positions, much like the defense industry and the Pentagon. What this means is that healthcare thought leadership tends to be in the hands of individuals who have internalized the high costs of AMCs and lack any cost-containment perspective. Dominated by physicians and thought leaders coming from AMCs and their closely related medical schools, there is a lack of knowledgeable high-level government leadership concerned with and focused on lowering hospital and healthcare costs; the past focus among US medical academia has always been on improved access to healthcare through greater funding, and only very rarely focusing on lower costs. One way to summarize the political situation is when it comes to current US healthcare costs, “the fox is guarding the henhouse.”

## Conclusions

We present a patient with a biopsy-confirmed benign lichenoid keratosis and 10 actinic keratoses that were successfully diagnosed and managed by a faculty member of a renowned academic medical center. The charges for delivering identical care would have been substantially lower in nearly all other healthcare delivery systems. If America’s storied AMCs are truly committed to living up to their lofty aspirations to eliminate healthcare disparities, hospital costs and charges should become a primary focus of their leadership.
